# Uncovering a novel mechanism: Butyrate induces estrogen receptor alpha activation independent of estrogen stimulation in MCF-7 breast cancer cells

**DOI:** 10.1590/1678-4685-GMB-2023-0110

**Published:** 2024-03-08

**Authors:** Veronica Dayali Gutierrez-Martinez, Alfonso León-Del-Río, Abelardo Camacho-Luis, Victor Manuel Ayala-Garcia, Angélica María Lopez-Rodriguez, Estela Ruiz-Baca, Ivan Meneses-Morales

**Affiliations:** 1 Universidad Juárez del Estado de Durango, Facultad de Ciencias Químicas, Durango, México.; 2 Universidad Nacional Autónoma de México, Instituto de Investigaciones Biomédicas, Ciudad de México, México.; 3 Universidad Juárez del Estado de Durango, Facultad de Medicina y Nutrición, Centro de Investigación en Alimentos y Nutrición, Durango, México.

**Keywords:** Butyrate, breast cancer, MCF-7, transcription, Estrogen Receptor

## Abstract

Butyrate is a promising candidate for an antitumoral drug, as it promotes cancer cell apoptosis and reduces hormone receptor activity, while promoting differentiation and proliferation in normal cells. However, the effects of low-dose butyrate on breast cancer cell cultures are unclear. We explored the impact of sub-therapeutic doses of butyrate on estrogen receptor alpha (ERα) transcriptional activity in MCF-7 cells, using RT-qPCR, Western blot, wound-healing assays, and chromatin immunoprecipitation. Our results showed that sub-therapeutic doses of sodium butyrate (0.1 - 0.2 mM) increased the transcription of ESR1, TFF1, and CSTD genes, but did not affect ERα protein levels. Moreover, we observed an increase in cell migration in wound-healing assays. ChIP assays revealed that treatment with 0.1 mM of sodium butyrate resulted in estrogen-independent recruitment of ERα at the pS2 promoter and loss of NCoR. Appropriate therapeutic dosage of butyrate is essential to avoid potential adverse effects on patients’ health, especially in the case of estrogen receptor-positive breast tumors. Sub-therapeutic doses of butyrate may induce undesirable cell processes, such as migration due to low-dose butyrate-mediated ERα activation. These findings shed light on the complex effects of butyrate in breast cancer and provide insights for research in the development of antitumoral drugs.

## Introduction

Breast cancer is a significant global health concern, with 70% of breast tumors being estrogen-dependent (Meneses-Morales et al., 2014). While tamoxifen therapy is an effective treatment for hormone receptor-positive breast cancer in premenopausal women, prolonged administration can lead to tumor resistance and increase the risk of developing bone and uterus cancer (Barrios-García et al., 2014). Currently, efforts are focused on developing improved antitumoral strategies to combat human breast cancer.

Short-chain fatty acids (SCFAs) have been found to play roles in epigenetic regulation (Fellows and Varga-Weisz, 2020). Butyrate, a SCFA produced by the intestinal fermentation of dietary fiber by associated microbiota (Louis and Flint, 2017), has been the subject of significant research due to the “butyrate paradox,” which describes the differential effects of treatment on normal and tumor cells (Donohoe et al., 2012). In particular, butyrate treatment at doses over 2 mM acts as a carbon source in colonocytes but drives apoptosis mechanisms in tumor colon cells (Berni Canani et al., 2012). Additionally, butyrate has been shown to induce a decrease in the expression of estrogen, progesterone, and prolactin receptors (DeFazio et al., 1992; Ormandy et al., 1992; Hamer et al., 2008). These findings suggest that butyrate may hold promise as a potential therapeutic agent for breast cancer treatment.

Butyrate is a promising agent for treating cancer, particularly hormone receptor-dependent cancers such as breast cancer (Chen et al., 2019 b ; He et al., 2021; Jaye et al., 2022). Studies have shown that butyrate can enhance the efficacy of established therapies such as doxorubicin, irinotecan, or oxaliplatin when used as an adjuvant (Chen *et al.*, 2019a; He *et al.*, 2021). However, the clinical use of butyrate is limited by its rapid metabolization in the liver and enterocytes following oral or rectal administration, resulting in poor plasma concentrations that are lower than therapeutic requirements (Davis et al., 2000; Blaak et al., 2020). To address this limitation, new delivery systems are currently under development to achieve stable plasma concentrations of butyrate (Roda et al., 2007; Donovan et al., 2017; Wang et al., 2023).

The intestinal microbiota is the primary source of short-chain fatty acids, and the butyrate concentration in the colon ranges from 14.7 to 24.4 mM (Salimi et al., 2017; Blaak et al., 2020). In contrast, plasma concentrations are typically less than 20 μM (Olsson et al., 2021; Martinsson et al., 2022; Tang et al., 2022). Experimental conditions demonstrating butyrate’s antitumoral activity typically involve at least 2 mM (Meneses-Morales et al., 2019). However, subtherapeutic concentrations of butyrate (less than 0.5 mM) have received little attention in antitumoral research due to their perceived lack of anticancer action. Nonetheless, previous reports have shown that treatment with less than 0.5 mM of butyrate can induce ligand-independent transcription of prostatic-specific antigen in a prostate cancer cell line (Sadar and Gleave, 2000) and induce estrogen receptor alpha mRNA in a breast cancer cell line treated with a concentration of 0.3 mM of butyrate (DeFazio et al., 1992). Another report showed increased proliferation of a colon cancer cell line treated with 0.5 mM of butyrate (Donohoe et al., 2012). 

Breast cancer is a complex and challenging disease to treat, and butyrate has emerged as a promising candidate for its antitumoral potential. However, subtherapeutic doses of butyrate are a plausible scenario in the clinical setting, and its effects on cancer cells are poorly understood. Thus, this study aimed to investigate the cellular responses to subtherapeutic doses of butyrate in a breast cancer cell line as a model. Our findings reveal that butyrate can activate hormone receptors, stimulate transcription of estrogen-dependent genes, and promote migration of breast cancer cells. By elucidating the effects of low-dose butyrate treatment on breast cancer cells, we can better understand the mechanisms underlying butyrate’s antitumoral potential and optimize its clinical use for breast cancer treatment.

## Material and Methods

Cell culture and Treatment

The MCF-7 breast cancer cell line, representative of the luminal A subtype and characterized by the expression of estrogen receptor (ER) and progesterone receptor (PR), was procured from ATCC (Manassas, VA) by the Instituto de Investigaciones Biomédicas, UNAM. Subsequently, it was graciously provided to the Facultad de Ciencias Químicas, UJED. Cultivation of MCF-7 cells was carried out in DMEM medium supplemented with 10% FBS, antibiotics, and antimycotic agents until reaching confluence. The cells were then seeded in six-well plates and, after 24 hours, were washed with PBS and maintained in DMEM without phenol-red and 10% charcoal-stripped FBS for 4 days to reach hormone deprivation conditions. To study the effects of butyrate on MCF-7 cells sodium butyrate (NaB) was purchased from Sigma-Aldrich (St. Louis, MO, USA), five different concentrations (0.1 to 2 mM) and one without treatment (control condition) were used. The cells were treated with sodium butyrate for 16 hours and then harvested for further analysis.

RNA Isolation and Real-time PCR

Total RNA was extracted from the harvested cells using RNA-Get (BioTecMol). Retrotranscription reactions were performed using 2 µg of total RNA, oligo (dT) primer, hexamer mix, and SuperScript III (Invitrogen). Real-time PCR reactions were performed using Amplificasa Taq-polymerase (BioTecMol), EvaGreen, and ROX, with specific primers designed by PrimerQuest IDT-software for ACTB, ESR1, TFF1, and CTSD, which spanned exon junctions and were optimized for intercalating dye fluorescence detection in the QuantStudio 3 PCR machine; Primer sequences were: 5’-CGGCATTCTACAGGCCAAATTCAG-3’ (forward) and 5’-CTTCTCTTGAAGAAG GCCTTGCAG-3’ (reverse) for ESR1; 5’-CTGATTCAGGGCGAGTACAT-3’ (forward) and 5’-GACACCTTGAGCGTGTAGT-3’ (reverse) for CTSD (Cathepsin D); 5’-CCCT CCCAGTGTGCAAATAA-3’ (forward) and 5’-AAATTCACACTCCTCTTCTGGAG-3’ (reverse) for TFF1 (pS2); 5’-GGCACCACACCTTCTACAAT-3’ (forward) and 5’-AAC ATGATCTGGGTCATCTTCTC-3’ (reverse) for ACTB (b-actin) mRNA. The mRNA levels were calculated using the comparative Ct method and expressed as a fold increase relative to the control condition after normalization using beta-actin gene expression levels.

Western blotting

MCF-7 cells were seeded in p100 plates and incubated with five sodium butyrate treatments (0.1 to 2 mM) and one control condition for 24 and 48 hours. The cells were then harvested and lysed using Triton x-100 buffer plus 2 mM sodium decavanadate pH 7.6 to release nuclear receptors from chromatin. The protein extracts were quantified using the Bradford method. 30 micrograms of each total protein extract were loaded onto SDS-PAGE gels, transferred to PVDF membranes, and incubated overnight with primary antibodies against beta-actin and estrogen receptor alpha (Santa Cruz, CA). The proteins were visualized using a secondary horseradish-peroxidase-conjugated antibody and an enhanced chemiluminescence (BM Chemiluminescence Western Blotting Kit (Mouse/Rabbit), Roche). The results were digitalized using a ChemiDoc Bio-Rad® gel imaging system.

Wound-healing assay

MCF-7 cells were seeded in 6-well dishes. After confluence, the monolayer was “scratch-wounded” in triplicate, washed with PBS and treated with five sodium butyrate treatments (0.1 to 2 mM) and one control condition. Images of the cells were captured at the beginning and every 24 hours for three days to monitor cell migration and wound closure. The migration rate of the cells was quantified using ImageJ and Fiji plugin.

Chromatin immunoprecipitation

To investigate the binding of estrogen receptor alpha (ERα) to the pS2 gene promoter in response to butyrate and estradiol treatments, we performed chromatin immunoprecipitation (ChIP) assays. MCF-7 cells were treated with sodium butyrate, or a control condition for 45 min, crosslinked with formaldehyde, and sonicated to fragment the chromatin. Then, 2 mg of specific anti-ERα antibody or anti-luciferase as a control antibody was added to two mg of chromatin extract, and the mixture was incubated overnight at 4°C. We used a DNA region located 3 kb upstream of the pS2 promoter as a negative control. After immunoprecipitation, the DNA-protein complexes were eluted, reversed crosslinked, and purified. The pS2 gene promoter region and the control region were amplified by PCR using the immunoprecipitated chromatin as a template; the primers sequences were: 5’-CCG GCCATCTCTCACTATGAA-3’ (forward) and 5’-GGTCATCTTGGCTGAGGGATCT-3’ (reverse) for pS2 promoter region; 5’-AGCTGGGTGTCCTTGTAAAG-3’ (forward) and 5’-AGTTT GGGAGGAAGTGGATC-3’ (reverse) for pS2 control control. The PCR products were separated on a 2.5% agarose gel, visualized with GelRed, and quantified by densitometry analysis using the ChemiDoc gel imaging system and Quantity One software (Bio-Rad).

Statistical analysis

All experiments were performed as independent triplicates, and the results are expressed as the mean ± standard error of the mean. Statistical significance was assessed utilizing Student’s *t*-test or ANOVA, with a predetermined significance level of 0.05, as outlined in the figure legends. Data analysis was carried out using the OriginPro 2021 statistical software.

## Results

**Figure 1-:**
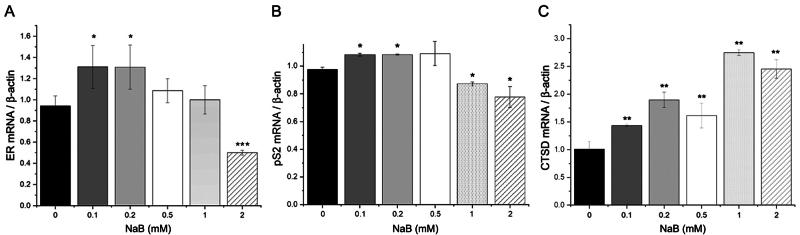
Subtherapeutic doses of sodium butyrate (NaB) can enhance estrogen receptor-mediated transcription in a ligand-independent manner. The results demonstrate the RT-qPCR assessment of mRNA expression for ERα (A), pS2 (B), and Cathepsin D (C) in MCF-7 cells after a 16-hour treatment with butyrate. The data were normalized to beta-actin, and the experiment was repeated three times (*p<0.05).

Treatment with subtherapeutic doses of sodium butyrate (0.1- and 0.2 mM) increased the expression of estrogen receptor alpha (ERα) and estrogen-responsive genes pS2 and Cathepsin D in MCF-7 cells, as measured by RT-qPCR (Figure 1). The ERα transcript was upregulated by 30% with low-dose sodium butyrate treatment (Figure 1 A), while pS2 by 20% and Cathepsin D as much as 80% (Figure 1 B  and 1C). These findings suggest that low-dose butyrate induces estrogen-independent ERα transcriptional activity in MCF-7 cells. As previously reported, the administration of a therapeutic dose of butyrate (>2 mM) resulted in a decrease in the expression of ERα and pS2 transcripts.

**Figure 2-:**
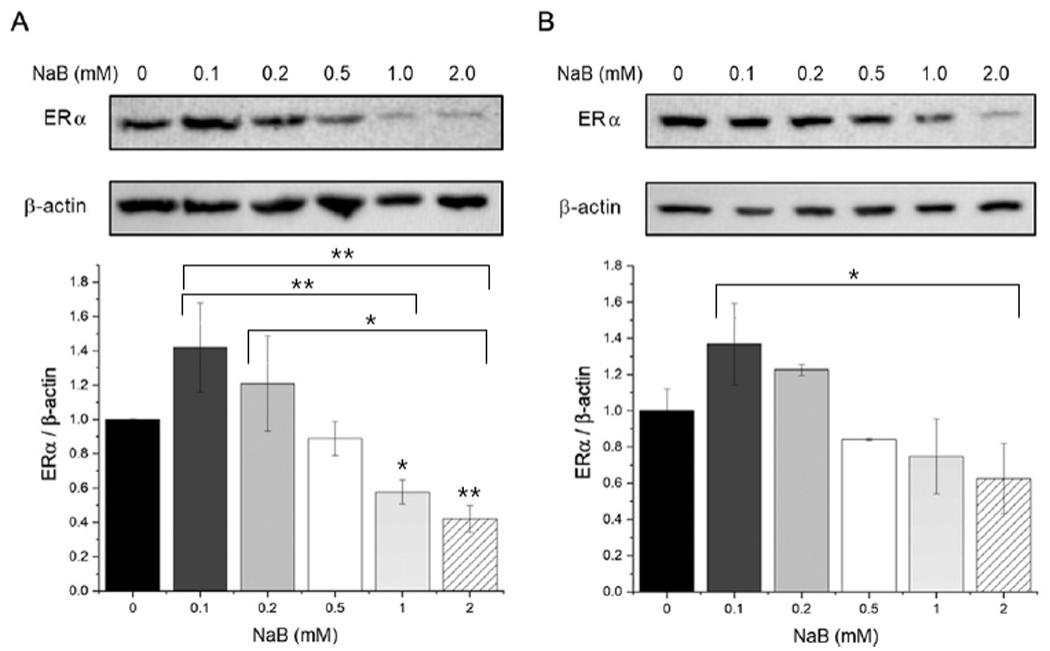
High doses of sodium butyrate (NaB) significantly decrease the levels of estrogen receptor protein. Western blot analysis of ERα protein after 24 h (A) or 48 h (B) of treatment did not reveal any statistically significant increase in response to 0.1- and 0.2-mM concentrations of sodium butyrate. However, higher concentrations of NaB led to a decrease in ERα protein signal (n = 3; *p<0.05; **p<0.01).

The effects of subtherapeutic doses of butyrate on ERα protein expression were investigated in MCF-7 cells using western blot analysis. After treatment for 24 and 48 hours, a slight increase in ERα protein expression was observed beyond 24 hours (Figure 2 A  and 2B). However, these differences were not statistically significant. On the other hand, treatment with higher doses of sodium butyrate (≥1 mM) resulted in a decrease in ERα protein expression, which is consistent with previous reports.

**Figure 3 -:**
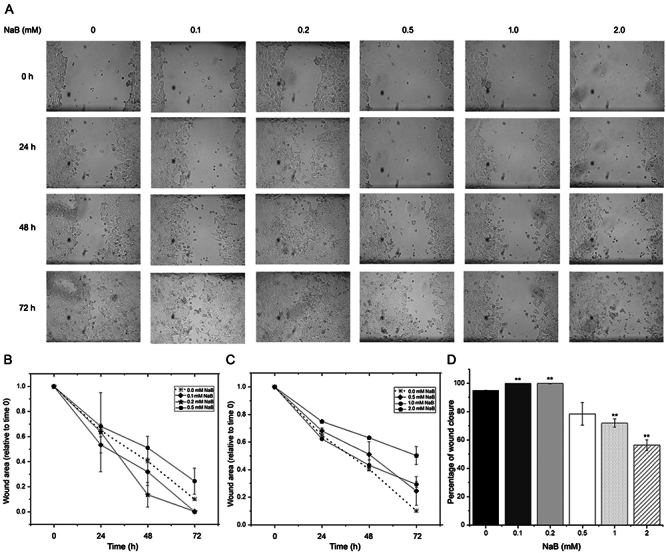
Subtherapeutic doses of sodium butyrate (NaB) significantly increased cell migration as evaluated through the “scratch-wound” healing assay. The results obtained at different time points (A) with subtherapeutic (B), and therapeutic (C) doses of NaB showed differential effects, as demonstrated by the percentage of wound closure observed after the 72-hour assay (D). The data were normalized to the control condition (n=3; **p<0.01).

Previous studies have suggested that estrogen receptor ligands such as tamoxifen can modulate cell migration (Lymperatou et al., 2013; Sabol et al., 2014; Han et al., 2018). To investigate whether estrogen-independent activation of estrogen receptor by subtherapeutic doses of butyrate can influence cell migration, we performed wound-healing assays in MCF-7 cells treated with different concentrations of NaB (Figure 3 A ). As shown in Figure 3 B, treatment with 0.1 and 0.2 mM of sodium butyrate led to a faster wound-area reduction compared to the control condition, indicating enhanced cell migration. In contrast, higher doses of sodium butyrate (≥1 mM) did not induce wound-area reduction (Figure 3 C ), suggesting that the effect on migration is specific to subtherapeutic doses of butyrate. Consistent with these findings, 72 h wound-healing assays revealed a significant increase in wound closure with subtherapeutic doses of butyrate compared to therapeutic ones (Figure 3 D ).

**Figure 4 -:**
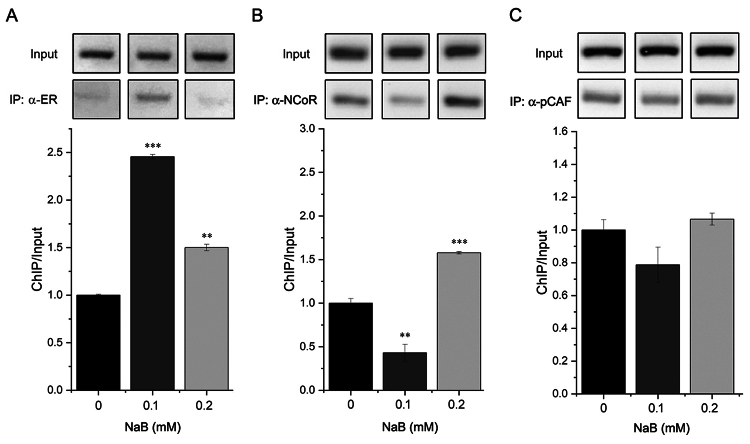
Butyrate induces ligand-independent recruitment of the estrogen receptor to the pS2 promoter. We performed pS2 promoter-specific PCR and used total chromatin as a positive control for Input (5%) amplification (Up) and antibody-precipitated chromatin from MCF-7 cells treated with sodium butyrate as a template (Down). We performed triplicate experiments and generated graphs to show the recruitment of estrogen receptor alpha (A), NCoR (B), and pCAF (C) under different sodium butyrate (NaB) treatments (n = 3; **p<0.01; ***p<0.001), using densitometry analysis.

To investigate the underlying mechanisms of butyrate-induced estrogen receptor activation, we performed chromatin immunoprecipitation assays to evaluate whether butyrate activates ERα through genomic mechanisms. Our results showed that treatment with 0.1 mM of sodium butyrate for 45 minutes led to estrogen receptor alpha-enriched recruitment at the pS2 promoter region, and to a lesser extent, with 0.2 mM (Figure 4 A ). We further investigated the effect of butyrate treatment on co-regulator recruitment at the pS2 promoter by performing ChIP assays with NCoR and pCAF antibodies. Our results showed a loss of binding of the transcriptional co-repressor NCoR to the pS2 promoter with 0.1 mM of sodium butyrate treatment and an increased binding with 0.2 mM (Figure 4 B ). In contrast, our assays with MCF-7 cells under the conditions of 0.1 and 0.2 mM of NaB for 45 minutes showed no significant statistical differences in co-activator pCAF recruitment (Figure 4 C ). We used an anti-luciferase antibody for the control chromatin immunoprecipitation, and PCR control reactions with primers specific to a region three kb upstream of the pS2 promoter as recruitment-negative control did not yield amplification products (not shown).

Taken together, our findings demonstrate that subtherapeutic doses of butyrate can activate estrogen receptor-mediated transcription and enhance cell migration in MCF-7 cells. Our chromatin immunoprecipitation assays suggest that these effects may be mediated through genomic mechanisms involving estrogen receptor alpha recruitment and co-regulator binding as for the pS2 promoter. These results provide new insights into the potential role of butyrate in modulating estrogen receptor signaling in breast cancer.

## Discussion

In this study, we investigated the influence of subtherapeutic doses of butyrate on ERα activity and its cellular implications. Our chromatin immunoprecipitation assays showed that subtherapeutic doses of butyrate induce estrogen independent ERα transcriptional activity, such as for the enhanced ERα recruitment to the pS2 promoter region. This finding is significant because it reveals a previously unknown mechanism by which butyrate regulates estrogen receptor activity.

Previous studies have investigated the effects of butyrate treatment on gene expression in various cancer cell lines. For example, Sadar and colleagues (2000) reported on the role of butyrate in regulating the expression of prostate-specific antigen (PSA) in LNCaP prostate cancer cells. They discovered that low concentrations of butyrate (0.2-0.5 mM) increased PSA mRNA levels, while higher concentrations (0.5-5 mM) decreased its expression. Furthermore, their results suggested that butyrate could activate androgen receptor (AR) transactivation activity in a ligand-independent manner. Our study using real-time PCR revealed a statistically significant difference in the mRNA levels of ERα, pS2, and Cathepsin-D under low sodium butyrate treatments. Specifically, we observed an increase in the mRNA levels of pS2 and Cathepsin-D in MCF-7 cells treated with 0.1- and 0.2-mM sodium butyrate, which suggests that subtherapeutic doses of butyrate can induce ERα transcriptional activity. However, higher concentrations of butyrate were found to decrease the mRNA levels of ERα and pS2, consistent with previous reports (DeFazio et al., 1992; Sun et al., 2005), these actions could be linked to the HDAC inhibitor role of butyrate (Donohoe et al., 2012). In the case of Cathepsin-D mRNA, we observed a further increase in mRNA levels following treatment with 1 and 2 mM of NaB, which is likely due to the induction of apoptosis, as previously reported (Minarowska et al., 2007).

Although an increase in ERα mRNA levels was observed, western blot assays did not show any significant changes in ERα protein levels after 0.1- and 0.2-mM sodium butyrate treatments at 24 h or 48 h. However, higher concentrations of sodium butyrate resulted in a decrease in estrogen receptor protein levels, consistent with previous reports (DeFazio et al., 1992). These findings emphasize the multifaceted effects of butyrate on estrogen receptor regulation.

Our “wound-healing” assays revealed a significant increase in the speed of scratch closure in MCF-7 monolayers treated with subtherapeutic doses (0.1 and 0.2 mM) of sodium butyrate, indicating the potential of butyrate to induce collective cell migration. Prior investigations have consistently indicated an inhibitory impact of various concentrations of butyrate (ranging from 0.1 to 2 mM and higher) on the proliferation of MCF-7 and other breast cancer cell lines. This inhibition was determined through MTT or CCK-8 assays conducted over a 4-day period, with measurements recorded at 24-hour intervals (Li et al., 2015; Salimi et al., 2017). These findings were further substantiated in the context of a colon cancer cell line by Li *et al.* in 2018. The researchers replicated similar experiments utilizing HCT116 cells and the CCK-8 assay. As a result, these consistent findings reinforce the proposition that the observed enhancement in wound closure is more plausibly attributed to an augmentation in cell migration rather than the induction of cell proliferation. Future studies should investigate the impact of butyrate on ERα-negative cell lines, such as MDA-MB-231, to determine whether the effect of butyrate on cell migration is ERα-dependent or related to the enhanced histone acetyltransferase (HAT) activity induced by lower butyrate concentrations (Donohoe et al., 2012).

It is important to note that the concentration of butyrate in plasma is typically less than 20 μM (Olsson et al., 2021; Martinsson et al., 2022; Tang et al., 2022). In order to achieve antitumoral effects, concentrations higher than 2 mM are typically required (Meneses-Morales et al., 2019). Our results demonstrate a dual influence of butyrate concentration on estrogen receptor activity, indicating a narrow therapeutic window for butyrate. This suggests the necessity of a fine balance tuning between subtherapeutic concentrations and antitumoral effects.

According to previous reports, low-dose butyrate treatment has the potential to increase the availability of acetyl groups and activate histone acetyltransferases (HATs) (Donohoe et al., 2012). In this study, we sought to investigate whether our findings could be attributed to genomic mechanisms of regulation. To this end, we conducted chromatin immunoprecipitation assays and found that subtherapeutic doses of sodium butyrate (0.1 mM) led to an estrogen-independent recruitment of estrogen receptor alpha to the pS2 promoter in MCF-7 cells. These results suggest that low-dose butyrate treatment may induce ERα transcriptional activation through estrogen-independent mechanisms.

After examining the effects of butyrate on the recruitment of representative nuclear receptor co-regulators, NCoR and pCAF, our study did not yield conclusive evidence. Consequently, the precise mechanisms by which butyrate facilitates the recruitment of nuclear hormone receptors to their regulated promoters’ cognate sequence remain unclear. To gain a better understanding of these mechanisms, it is essential to conduct additional research that considers the temporal dynamics of co-regulator recruitment under butyrate treatment. Such research would help to clarify the molecular pathways involved in butyrate-induced co-regulator recruitment and its downstream effects on nuclear hormone receptor activity at regulated promoters.

The current study has some limitations that should be acknowledged. Firstly, we employed a single-cell line (MCF-7) to examine the impact of butyrate on ERα transcriptional regulation. Although MCF-7 is a well-established cellular model for estrogen receptor-dependent breast cancer, this choice may constrain the generalizability of our results. Future investigations could broaden the scope of our findings by exploring the effects of butyrate on various other cell lines. Such efforts would provide a more comprehensive understanding of the potential applications and limitations of butyrate as a treatment for breast cancer.

To summarize, our study adds to the expanding body of research on the influence of butyrate on gene expression and underscores the potential therapeutic risks of butyrate in cancer treatment. Our findings demonstrate that even subtherapeutic doses of butyrate can elicit estrogen-independent ERα transcriptional activity, which could have significant implications for treating estrogen receptor-positive breast cancer. These results indicate that butyrate has the potential to be a valuable addition to existing breast cancer therapies, nonetheless, additional studies are needed to further understand the mechanistic underpinnings of butyrate’s effects on ERα transcriptional regulation and to optimize its potential for clinical use in treating breast cancer.
